# Determination of serum tumor necrosis factor-alpha (TNF-α) levels in metabolic syndrome patients from Saudi population

**DOI:** 10.12669/pjms.37.3.3897

**Published:** 2021

**Authors:** Muhammad Ikram Ullah, Badr Alzahrani, Abdullah Alsrhani, Muhammad Atif, Ayman Ali Mohammed Alameen, Hasan Ejaz

**Affiliations:** 1 Muhammad Ikram Ullah, PhD., Department of Clinical Laboratory Sciences, College of Applied Medical Sciences, Jouf University, Al Jouf, Saudi Arabia; 2 Badr Alzahrani, PhD., Department of Clinical Laboratory Sciences, College of Applied Medical Sciences, Jouf University, Al Jouf, Saudi Arabia; 3 Abdullah Alsrhani, PhD., Department of Clinical Laboratory Sciences, College of Applied Medical Sciences, Jouf University, Al Jouf, Saudi Arabia; 4 Muhammad Atif, M.Phil., Department of Clinical Laboratory Sciences, College of Applied Medical Sciences, Jouf University, Al Jouf, Saudi Arabia; 5 Ayman Ali Mohammed Alameen, PhD., Department of Chemical Pathology, Faculty of Medical Laboratory Sciences, University of Khartoum, Sudan; 6 Hasan Ejaz, PhD.. Department of Clinical Laboratory Sciences, College of Applied Medical Sciences, Jouf University, Al Jouf, Saudi Arabia

**Keywords:** Metabolic syndrome, Inflammatory markers, Serum concentrations, TNF-α, Saudi population

## Abstract

**Objectives:**

To detect the relationship between serum tumor necrosis factor-alpha (TNF-α) and metabolic syndrome (MetS) components in patients of the Saudi population.

**Methods:**

This cross-sectional study was carried out at Jouf University Saudi Arabia from September 2019 to August 2020 and comprised of 183 individuals (91 cases and 92 controls). The blood samples were drawn from the patients visiting two tertiary care settings of Al Jouf province. Biochemical analysis was conducted on various instruments, and serum TNF-α was measured by the ELISA method.

**Results:**

The levels of serum glucose fasting, lipid profile, HbA1c and body mass index (BMI) were raised significantly in cases of MetS than controls (p = 0.001). Serum TNF-α was significantly higher in patients (58.04 ± 15.44) than controls (48.81 ± 10.30). It was correlated with the BMI, blood HbA1c, serum fasting glucose (SFG) and serum high density lipoprotein (HDL). The weak positive correlation was found with BMI (r = 0.18; p = 0.01), serum glucose (r = 0.21; p = 0.007) and HbA1c (r = 0.14; p = 0.04), but found negative association with serum HDL (r = -0.18; p = 0.01).

**Conclusion:**

The serum TNF-α was raised in metabolic syndrome patients than the healthy controls. It was positively associated with high BMI, serum fasting glucose, and HbA1c and found linked and negatively linked to low HDL levels in MetS patients in the Saudi population.

## INTRODUCTION

The metabolic syndrome (MetS) or syndrome X is a devastating disorder augmented by lifestyle and obesity. Various characteristics contribute to this ailment, including the development of the cardiovascular disease, hypertension, impaired glucose tolerance, type 2 diabetes mellitus (T2DM), or impaired fasting hyperglycemia), insulin resistance, central obesity, and impaired lipid levels.^1^,^2^ These features may occur individually by chance, but they can raise cardiac risk in combination.^3^ As the metabolic syndrome is a complex anomaly, the definition for disease categorization has been explained by various organizations like Adult Treatment Panel III (ATP III), National Cholesterol Education Program (NCEP), the World Health Organization (WHO), and International Diabetes Foundation (IDF)and.^4^

Cytokines are immune mediators, which are chemically complex peptides that perform various regulatory functions, and their role has been connected to MetS and its different constituents.^5^ In the MetS, the excessive deposition of fat in adipose tissue provides an inflammation environment development triggered by the intrusion of various cells like lymphocytes, macrophages, and suppressor cells. Innate immunity activation results in the production of like tumor necrosis factor-alpha (TNF-α), create a connection between the inflammation and the components of MetS like central adiposity, insulin resistance (IR), and different other impediments.^6^

Previous studies have reported a significant link of TNF-α with the IR onset in metabolic syndrome. The synthesis of TNF-α in adipose tissue reflects the direct connection between TNF-α serum levels, obesity, and hyper-insulinemia.^7^ Also, it is considered that TNF-α can control a vital regulator like autoparacrine glands for the fat cell to scrutinize the expansion of adipose-tissue, possibly by the inducing mechanism of insulin resistance, which can further lead to metabolic syndrome.^8^ The present study’s objective was to determine the serum TNF-α concentrations in Saudi patients of MetS and the normal controls and to detect their relationship with different MetS components.

## METHODS

Prior to the study’s start, ethical approval was taken from the Ethical Committee of Jouf University (Reference; HAP-13-s-001). Informed consent was taken from all patients and control subjects to enroll in the study.

This cross-sectional study was conducted out at Jouf University Saudi Arabia from September 2019 to August 2020. A total of 183 subjects, including 91 patients and 92 healthy controls, were enrolled with a convenient sampling technique from the Al Jouf region of Saudi Arabia. The patients were registered from the King Abdul Aziz Specialist Hospital and Prince Mutab Bin Ameer Hospital Sakaka Al Jouf. Guidelines of the International Diabetes Foundation (IDF) were used to establish MetS. Inclusion criteria of the patients with three of the following characteristics was based; **a**) the waist circumference of ≥90 cm in males and ≥80 cm in females, **b**) serum levels of triglycerides (TGS) less than 150 mg/dL or on treatment for TGs, **c**) if serum HDL is <40 mg/dL in males and <50 mg/dL in women or individuals on anti-dyslipidemia treatment **d**) if systolic blood pressure (SBP) >130 and diastolic blood pressure >85 or patients on anti-hypertensive treatment **e**) the level of fasting blood glucose (FBG) >100mg/dL or diabetic patients on anti-hyperglycemic treatment. The patients with different ailments, including hepatic decompensating, renal failure, acute and chronic infections, inflammation, and secondary causes of diabetes mellitus were excluded from this study. Demographic data was collected for all the participants through a predesigned proforma about height, weight, familial diabetic history, age, and gender. The body mass index (BMI) calculation was carried out by dividing body weight to height (kg/m2). All the control subjects had no history of familial diabetes and no history of taking insulin, and their BMI was normal.

Venous blood was collected aseptically from the individuals after an overnight fast of 10-12 hours in an EDTA vacutainer for HbA1c and serum separating vacutainer for biochemical analyses. A centrifuge was used to separate the serum at 5000 rpm for 10 minutes. After the centrifugation, serum was removed and transferred in the eppendorf tube (1.5 mL) for further analyses.

Biochemical tests were performed according to the guidelines provided by commercially available protocols. Serum glucose fasting, serum lipid profile were performed by Clinical chemistry Analyzer (Mindray BS-300). Blood HbA1c was performed by HPLC methods according to the standard protocols.

The serum TNF-α was separated from the whole blood, and the ELISA technique measured levels of TNF-α in all the participants. Commercially available ELISA kits were purchased, containing the pre-coated microtiter plate with a specific antibody to TNF-α standards. The pack included washing buffers and conjugates containing horseradish peroxidase (HRP) for TNF-α (Bioassay Technology Laboratory Co., Ltd, Shanghai, China). The optical density was measured at 450 nm by the automated microplate reader (Elsys Quattro, Human).

Data was analyzed using SPSS version 23. An independent student t-test was applied to calculate the mean (mean ± SD) difference between the subjects, including controls and cases. Bivariate correlation analysis was done to see the relationship among different variables. The association of serum TNF-α levels with serum glucose, lipid profile, HbA1c, and BMI was analyzed by Pearson’s correlation coefficient (r). The p-value was considered statistically significant if ≤ 0.05.

## RESULTS

A total of 183 study participants were enrolled in the Al Jouf region of Saudi Arabia. Out of total individuals, about 91 were metabolic syndrome (MetS) patients, and 92 were health controls without the components of MetS. Serum levels of the biochemical profile and the serum TNF-α were calculated in all recruited individuals.

The comparison of demographic and biochemical parameters was made between the MetS cases and healthy controls. The mean (mean ± SD) of age in the patients was 56.40 ± 11.96 years, and for the healthy controls, it was 56.79 ± 10.96 with no significant relation (p = 0.81). The mean BMI was lower in controls (27.20 ± 3.51) than MetS cases (34.20 ± 3.44), which was statistically significant (p = 0.04). The comparison of the biochemical parameters, including the serum fasting glucose, lipid profile, and HbA1c levels, was significantly raised in MetS cases compared to the controls presented in Table-I. The mean of fasting glucose in the patients and the controls was 187.60 ± 57.60 and 100.82 ± 11.40, respectively (p < 0.001). The serum lipids levels, including total serum cholesterol, serum triglyceride, and serum LDL, were raised significantly in patients with MetS in comparison to the normal controls (p < 0.05). In contrast, the serum HDL was decreased in patients than the controls with a significant difference (p = 0.001). The mean levels of blood HbA1c were found significantly high in the MetS cases compared to the healthy controls (p = 0.003). The mean serum TNF-α was higher (58.04 ± 15.44) in patients than in controls (48.81 ± 10.30), which was considered statistically significant with a p-value of 0.003.

**Table-I:** Comparison of demographic and descriptive data between metabolic syndrome patients and healthy controls (n=183)

Variables	MetS Cases (n=91) Mean ± SD	Normal controls (n=92) Mean ± SD	t-test	p-values
Age (years)	56.40 ± 11.96	56.79 ± 10.96	0.636	0.81
BMI (cm^2^)	34.20 ± 3.44	27.20 ± 3.51	-2.016	0.04*
Serum Fasting Glucose (mg/dL)	187.60 ± 57.60	100.82 ± 11.40	3.35	< 0.001*
Total Cholesterol (mg/dL)	230.60 ± 23.50	173.50 ± 23.90	1.94	0.05*
Triglyceride (mg/dL)	197.85 ± 53.73	129.42 ± 32.34	4.78	< 0.001*
LDL (mg/dL)	175.42 ± 28.64	117.81 ± 31.45	4.11	< 0.001*
HDL (mg/dL)	42.63 ± 7.92	58.26 ± 11.72	3.71	< 0.001*
HbA1c (%)	8.01 ± 2.22	5.13 ± 0.63	5.208	< 0.001*
TNF- α (pg/mL)	58.04 ± 15.44	48.81 ± 10.30	2.981	0.003*

* p-value < 0.05 was considered statistically significant.

**Fig.1:**
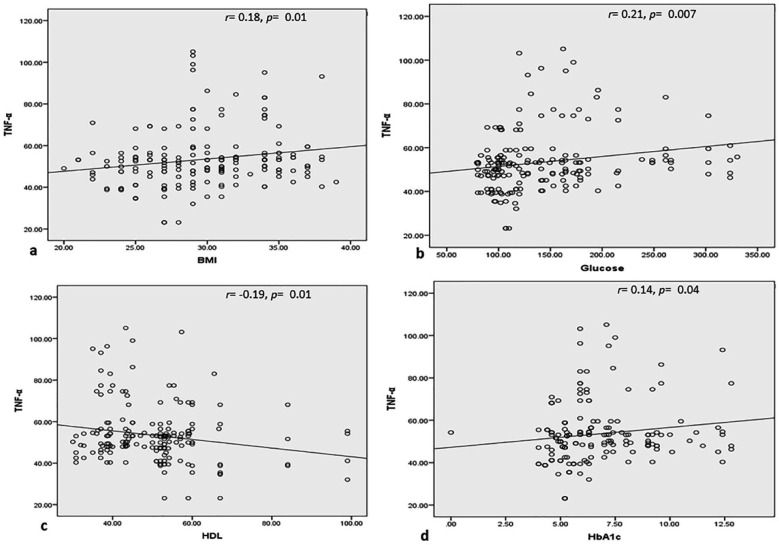
The correlation of serum TNF-α levels (pg/mL) with BMI (a), serum glucose (b), serum HDL (c), and blood HbA1c (d) in metabolic syndrome patients.

For non-normally-distributed variables, non-parametric tests of Pearson correlation were applied. A weak positive link was detected between serum fasting glucose and BMI in the patients’ group; serum lipid profile and blood HbA1c were positively correlated (p = 0.001). On the other hand, the serum HDL-cholesterol was weak negatively correlated with different biochemical profiles and BMI (p < 0.05) in MetS as shown in Table-II. In MetS patients, serum TNF-α concentration was positively associated with the BMI (r = 0.18), serum fasting glucose (r = 0.21), and blood HbA1c (r = 0.14) and has a negative link with the serum HDL (r = -0.19) with a p-value < 0.05 demonstrating an important relation with different components of MetS presented in Fig.1.

**Table-II:** The correlation of different biochemical parameters in metabolic syndrome patients.

Variables	Statistics	Age	BMI	Glucose	Total Cholesterol	Triglycerides	LDL	HDL
Age (years)	PC	1						
	p-value							
BMI (kg/cm^2^)	PC	-0.02	1					
	p-value	0.80						
Glucose (mg/dL)	PC	0.01	0.41	1				
	p-value	0.87	0.001^*^					
Total Cholesterol (mg/dL)	PC	0.11	0.40	0.43	1			
	p-value	0.28	0.001^*^	0.001*				
Triglycerides (mg/dL)	PC	-0.06	0.41	0.44	0.41	1		
	p-value	0.37	0.001^*^	0.001^*^	0.001^*^					
LDL (mg/dL)	PC	-0.05	0.41	0.51	0.54	0.42	1	
	p-value	0.50	0.001^*^	0.001^*^	0.001^*^	0.001^*^			
HDL (mg/dL)	PC	0.04	-0.37	-0.43	-0.37	-0.36	-0.37	1
	p-value	0.61	0.001^*^	0.001^*^	0.001^*^	0.044^*^	0.002^*^	
HbA1c (%)	PC	0.01	0.45	0.40	0.43	0.42	0.45	-0.44
	p-value	0.88	0.001^*^	0.001^*^	0.001^*^	0.001^*^	0.001^*^	0.04^*^

* Significant Pearson correlation.

## DISCUSSION

Metabolic syndrome (MetS) results from the altered response of immunity and macrophage infiltration of adipose tissue. TNF-α is a pro-inflammatory cytokine secreted from the peri-visceral fat, and its role is linked to insulin resistance, atherosclerosis, and endothelial dysfunction.^2^,^9^ The role of inflammatory markers like TNF-α has not been widely documented in MetS, although its activity in MetS components is being described. Various studies have demonstrated that pro-inflammatory markers like TNF-α impart their roles in developing cardiovascular diseases, metabolic syndrome, obesity, type 2 diabetes, insulin resistance, and non-alcoholic fatty liver disease.^10^,^11^ The current study was carried out to demonstrate the association between serum TNF-α and MetS characteristics.

In the present study, it has been described that the concentration of serum TNF-α was high in MetS patients than the normal controls with a significant difference (p < 0.05). Serum TNF-α concentration also presented the correlation with HbA1c, serum lipid profile, serum fasting glucose, and BMI. A weak positive correlation of serum TNF-α was found between serum fasting glucose and BMI and HbA1c (p < 0.05). On the other hand, the serum HDL was negatively correlated in the patient group. Our results are comparable to the previous studies in which the different components of MetS are significantly associated with raised serum TNF-α.^12^,^13^

It has also been depicted that the high serum TNF-α associated with raised HbA1c in MetS may predict the glycemic control in these patients. In a previous study, the concentration of serum TNF-α was considerably raised in type 2 diabetes patients having high HbA1c levels.^14^ T2DM is considered an important component of MetS that can develop insulin resistance. Elevated serum TNF increases the development of risk forT2DM and poor glycemic control.^15^

On the other hand, high TNF-α expression in adipose cells results in obesity and insulin resistance progression.^16^ The mechanism of obesity development due to lipoprotein lipase (LPL) inhibition, leptin activity, and glucose homeostasis is triggered by TNF-α expression. It has been demonstrated that TNF-α expression has an important positive relation with leptin and BMI while having a substantial negative association with LPL activity, signifying that it may develop homeostasis that results in the fat deposition by the regulation of the activity of LPL and the formation of leptin.^17^

The high serum concentration of TNF-α is also documented in patients with overall MetS, rather than its components. In our study, high serum TNF-α is positively linked with the BMI and serum lipid profile. Simultaneously, it was negatively associated with serum HDL, which is consistent with the previous study in which the different components, including BMI, triglyceride, and obesity, were positively correlated while serum HDL was negatively correlated.^18^ On the other hand, some other studies documented that there was no correlation of serum TNF-α with different components of MetS, including serum lipid profile fasting blood sugar, obesity, and hypertension. Various reports have been documented to explore the relationship between high serum TNF-α expression with different components of impending metabolic syndrome.^19^,^20^

###  Limitations of the study:

It includes small sample size and literature regarding serum TNF in the Saudi population’s metabolic syndrome. Moreover, a group of inflammatory markers should be investigated to understand its role on a larger scale, which could not be achieved due to limited resources.

## CONCLUSION

The serum levels of TNF-α were found significantly higher in patients with MetS than healthy controls. Also, serum TNF-α concentrations were related positively with the serum fasting glucose, HbA1c, and different lipid profile components and negatively correlated with serum HDL.

## RECOMMENDATIONS

1. The results propose that inflammation could be an important factor for the disease’s predisposition.
2. It is suggested that serum TNF-α levels should be tested as a considerable risk factor for disease progression.

### Author’s Contribution:

**MIU:** Conceived the idea, performed analysis, manuscript drafting and is responsible and accountable for the accuracy or integrity of the work.

**BA:** Samples analysis and initial manuscript drafting.

**AA:** Sample collection, manuscript editing, and proofreading.

**MA:** Specimen analysis and results interpretation.

**AAMA:** Data collection and literature review.

**HE:** Clinical analysis and final editing of the manuscript.
